# Sepsis Management in the Cardiac Intensive Care Unit

**DOI:** 10.3390/jcdd10100429

**Published:** 2023-10-17

**Authors:** Yichi Zhang, Michael T. McCurdy, Jonathan Ludmir

**Affiliations:** 1 Department of Medicine, Massachusetts General Hospital, Boston, MA 02114, USA; yzhang@mgh.harvard.edu; 2 Division of Pulmonary & Critical Care, Department of Medicine, University of Maryland School of Medicine, Baltimore, MD 21201, USA; drmccurdy@gmail.com; 3 Corrigan Minehan Heart Center, Cardiology Division, Massachusetts General Hospital, Boston, MA 02114, USA

**Keywords:** sepsis, intensive care, cardiology, critical care, shock, ICU, CICU

## Abstract

Septic shock management in the cardiac intensive care unit (CICU) is challenging due to the complex interaction of pathophysiology between vasodilatory and cardiogenic shock, complicating how to optimally deploy fluid resuscitation, vasopressors, and mechanical circulatory support devices. Because mixed shock portends high mortality and morbidity, familiarity with quality, contemporary clinical evidence surrounding available therapeutic tools is needed to address the resultant wide range of complications that can arise. This review integrates pathophysiology principles and clinical recommendations to provide an organized, topic-based review of the nuanced intricacies of managing sepsis in the CICU.

## 1. Introduction

Sepsis and septic shock are common complications experienced by patients in the cardiac intensive care unit (CICU) [1]. The intricate pathophysiologic features and interactions between cardiogenic shock (CS) and septic shock crucially inform our current, multi-faceted diagnostic and therapeutic approach. This current literature review integrates relevant pathophysiology principles with guidance to manage patients with comorbid sepsis and CS.

## 2. Epidemiology and Definitions

The Third International Consensus defines sepsis as a “life-threatening organ dysfunction caused by a dysregulated host response to infection”. Organ dysfunction can be further qualified by a change in the sequential organ-failure assessment (SOFA) score of ≥2 points from baseline [2]. Though the truncated version of the SOFA score, the “quick SOFA” or “qSOFA” score, is easier to use by only accounting for respiratory rate (RR), mentation, and systolic blood pressure (SBP), it suffers from inferior prognostic accuracy compared to its traditional, more cumbersome counterpart [3]. Specifically, the qSOFA score alone should not be used to diagnose sepsis, but rather as an expedited prognostic tool for predicting mortality in patients with sepsis. Defined as worsening hemodynamics in the setting of sepsis, septic shock is characterized by the need for vasopressors to maintain a mean arterial pressure (MAP) of ≥65 mmHg and a serum lactate >2 mmol/L [4]. In this capacity, septic shock should specifically be considered in the absence of hypovolemia since infections can often illicit ongoing fluid losses (e.g., diarrhea or vomiting), resulting in a hypovolemic component to shock, which should be addressed first. 

In total, 6 to 37% of CICU patients experience sepsis [5,6], and up to 44% of those developing sepsis die [5]. Furthermore, patients with myocardial infarction (MI) complicated by sepsis experience 103% higher odds of death compared to non-septic MI patients [7]. Similarly, non-cardiac multisystem organ failure is also significantly more prevalent in sepsis cohorts.

Despite the absence of a standard definition for CS [6], one of the most well-known criteria originating from the SHOCK trial in 1999 defined it as (1) SBP < 90 mmHg for >30 min or a vasopressor need to maintain SBP ≥ 90 mmHg; (2) end-organ hypoperfusion (e.g., urine output < 30 mL/hour or cool extremities; and (3) cardiac index (CI) < 2.2 L/min/m^2^ and pulmonary capillary wedge pressure >15 mmHg [8]. The most recent European Society of Cardiology guidelines define CS similarly [9].

## 3. Pathophysiology of Cardiogenic and Septic Shock

Cardiogenic shock most commonly occurs in the setting of acute myocardial infarction (AMI), but can also occur due to decompensated HF, massive pulmonary embolism (PE), myocarditis, severe valvular dysfunction, and other causes [10]. In AMI-CS, the infarct results in reduced contractility and cardiac output (CO), which leads to increases in LV end-diastolic pressure (LVEDP). As left-side coronary perfusion depends on the difference between diastolic blood pressure and LVEDP, stiffer LV walls (and thus higher LVEDP) decrease coronary perfusion pressure (CPP), thus reducing aerobic respiration capabilities to initiate a vicious downward spiral towards systemic hemodynamic instability [11]. Unlike the LV, the RV can be perfused during both diastole and systole, though its CPP is still decided by the difference between MAP and RV pressure. The initial response of peripheral vasculature in the setting of cardiac dysfunction is to vasoconstrict to preserve perfusion pressure [12], and cells throughout the body maximize oxygen extraction from their respective capillary beds to compensate for reduced flow [13].

Sepsis results in peripheral circulatory vasodilatation through the action of nitric oxide (NO). Proinflammatory cytokines such as tumor necrosis factor (TNF) or interleukin-1 (IL-1) are upregulated as a result of the acute infection and inflammation, ramping up the production of NO, which diffuses through the circulation to activate guanylate cyclase [14]. The final product of this cascade, cyclic GMP, then relaxes vascular smooth muscle and inhibits vascular tone [15]. Dilated vessels thus can no longer maintain the perfusion pressure needed for optimal physiologic functioning, leading to widespread organ failure. Cardiac dysfunction can exist in up to 44% of patients presenting with septic shock [16]. Sepsis can worsen circulatory function by affecting either peripheral vasculature [14] or myocardial function [17].

Sepsis adversely impacts myocardial function directly via several distinct mechanisms. First, the aforementioned vasodilatory factor, NO, exerts inhibitory effects on beta-adrenergic (β_1_) receptors, which are normally responsible for increasing heart rate and contractility [17]. Decreased β_1_-receptor activity causes the heart to lose its compensatory reserve to combat shock. Second, sepsis-related mitochondrial dysregulation and subsequent reactive oxygen species (ROS) formation may also directly suppress cardiac function [18]. Myocardial cells are often saturated with mitochondria due to their high aerobic energy production needs. Thus, inflammatory cytokines that lead to mitochondrial dysfunction can lead to excessive ROS build-up and direct cytotoxic damage to the cardiomyocytes. Additionally, sepsis can induce complement system dysfunction, whereby complement factor C5a, a potent chemotaxis agent for mast cells and neutrophils, can directly suppress myocardial cell function [19]. Targeting these less-understood mechanisms of direct myocardial suppression can be a worthy pursuit in future biomedical or pharmaceutical research.

## 4. Hemodynamic Assessment and Diagnosis of Comorbid Sepsis and Cardiogenic Shock

Recognition and timely diagnosis of comorbid septic and cardiogenic shock in the CICU can be challenging. A comprehensive hemodynamic assessment is warranted to further understand (Table 1) the etiology of shock, whether cardiogenic, distributive, obstructive, or some combination. Comorbid septic and cardiogenic shock exhibit unique alterations in hemodynamic parameters (e.g., cardiac index, ventricular filling pressures, mixed venous oxygen saturation) that may exist in “paradoxical” combinations (e.g., coexisting preserved cardiac index with high filling pressures). Thus, in these complicated mixed shock scenarios, frequent and careful assessments of patients’ clinical status and hemodynamic parameters become even more necessary to inform proper diagnostics and ultimately guide management.

Cardiac biomarkers have been commonly obtained as part of the initial investigation for suspected acute cardiac dysfunction, including troponin and N-terminal pro-B-type natriuretic peptide (NT-proBNP) [6]. Specifically, troponin I, is often trended as a marker for myocardial injury in the setting of acute coronary syndrome, one of the most common etiologies for cardiogenic shock. Meta-analysis data also suggest the potential use of troponin I as a prognostic marker in septic shock, and there have been ongoing efforts to investigate this in large-scale, prospective trials [20,21]. NT-proBNP, on the other hand, is often used as a surrogate for estimating fluid status and cardiac congestion in the setting of heart failure. Similarly, in retrospective analysis, NT-proBNP has also been found to have prognostic value in septic shock [22]. However, a significant limitation of the utility of both troponin I and NT-proBNP in comorbid septic and cardiogenic shock lies in their specificity. Both markers are often found to be elevated in patients with chronic inflammatory conditions, as well as renal dysfunction, resulting in challenges with interpretation. Additionally, plasma renin has also been investigated as a biomarker for prognosis and treatment guidance in shock, with a comparison of its prognostic value against that of serum lactate. For example, a positive rate of change in plasma renin, but not lactate, for over 72 h has been associated with increased in-hospital mortality [23]. In a similar context, newer biomarkers, such as ST2 (a member of the interleukin receptor family) [24], copeptin (a molecule co-release with arginine vasopressin) [25], and growth differentiation factor 15 (GDF-15, a member of the transforming growth factor β superfamily) [26] are undergoing both retrospective and prospective investigations for their prognostic value in both cardiogenic and septic shock. However, their current clinical utility is greatly hindered by their limited availability and accessibility, which is heavily concentrated at large, academic clinical sites.

Two decades ago, a landmark trial showed mortality benefit from early goal-directed therapy (EGDT) involving hemodynamic monitoring of preload, afterload, and oxygen saturations with a central venous catheter in patients with septic shock [27]. Despite subsequent studies and meta-analyses not validating the benefits of deploying the specific EGDT protocol [27], close hemodynamic monitoring and the resultant timely appropriate resuscitative interventions have nevertheless been widely popularized in the intensive care setting. A wide variety of invasive and noninvasive tools and markers (e.g., end-tidal CO_2_, [ETCO_2_], inferior vena cava [IVC] collapsibility index as seen on point-of-care ultrasound [POCUS]) have been evaluated for their usefulness in hemodynamic monitoring in sepsis and CS [28]. Specifically, POCUS provides rapid and convenient global assessments of both LV and RV function, and it can be performed using remote guidance even in the most austere settings [29]. POCUS studies have explored the utility of venous doppler waveform analyses of the IVC alone, as well as composite analyses of the hepatic, portal, and renal veins to create the venous excess ultrasound score (VExUS), to help predict the severity of venous congestion [30,31]. Additionally, the rise of artificial intelligence-guided POCUS has the potential to reduce overall barrier-to-entry and increase inter-operator reliability for more skill-dependent measures such as the LV outflow tract velocity-time integral (VTI) [32]. However, the limitations of POCUS are equally important to recognize as it alone cannot replace a comprehensive cardiovascular assessment. Thus, POCUS findings must always be considered in the context of a physical exam and other clinical parameters to inform a more comprehensive picture of hemodynamic status. Additionally, perhaps the most straightforward method to assess fluid responsiveness can be achieved using a “passive leg raise”, which effectively delivers approximately 300 mL of preload to the heart [33]. This maneuver is safe due to its rapid reversibility and can be used as part of the initial hemodynamic assessment, even before test fluid boluses are given.

New advances in critical care technology have introduced tools to approach hemodynamic monitoring, such as pulse contour analysis, which gathers data from an arterial line to calculate cardiac output [34,35]. This technology has been validated against pulmonary arterial catheterization in stable patients undergoing surgery, yet its performance may be less reliable in clinical scenarios involving extremely low vascular resistance, such as sepsis and cirrhosis [35]. While these devices may account for the effects of fluid administration, their reliability should be balanced with a comprehensive clinical picture.

The current gold standard in hemodynamic assessment of cardiogenic shock remains the pulmonary artery catheter (PAC) [36]. Though invasive, the PAC can provide crucial, real-time guidance regarding left- and right-sided filling pressures and cardiac output to guide resuscitation decisions. Despite its ability to provide these hemodynamic data, many questions remain about its ability to translate those data into improved CS mortality, as suggested by a landmark meta-analysis conducted by Shah et al. in 2005 [37]. The authors concluded that perhaps the lack of benefit of PAC use resulted from a lack of “effective evidence-based treatments” used in conjunction with PAC [37]. However, in the past decade, advancements and tools in cardiac critical care have popularized the use of PACs for real-time monitoring of the therapeutic effect. Recent retrospective analyses suggest that the PAC is associated with lower propensity-matched 30-day mortality [38]. For example, PAC can characterize mixed shock profiles and can aid MCS-related clinical decision-making. Continually worsening hemodynamics may warrant escalating from an IABP to an Impella, adding left-ventricular assist device (LVAD) support to patients with only an RVAD, or initiating and up-titrating extracorporeal membrane oxygenation (ECMO) parameters [39]. While prospective, randomized control trials (RCTs) are needed, the PAC remains an invaluable tool to assist intensivists’ hemodynamic status assessment of complex CS patients.

## 5. Management Guidelines

**Figure 1:**
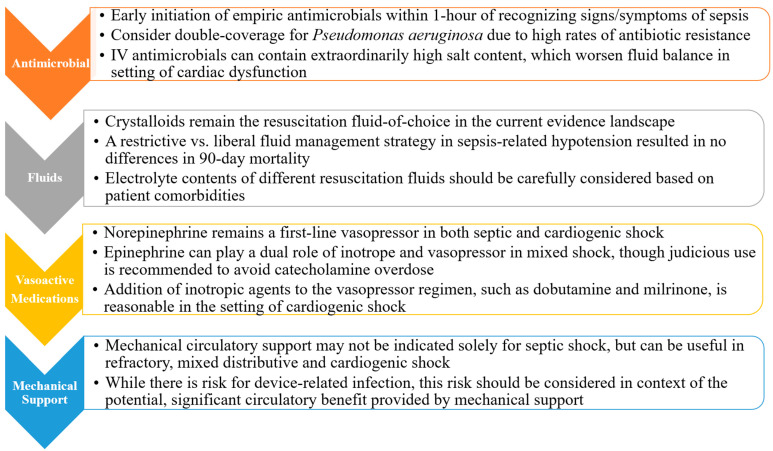
Stepwise Considerations in Management of Mixed Septic and Cardiogenic Shock.

Cardiogenic shock-related mortality in the CICU is significant, but comorbid septic shock adds an extra layer of complexity and risk that demands early recognition, careful monitoring, and a systematic approach to management (Figure 1).

### 5.1. Antimicrobials

Early empiric antimicrobial therapy is crucial for the treatment of sepsis and septic shock, preferably within one hour of recognizing the signs and symptoms [40]. Empiric antimicrobial agents are selected based on the most likely pathogen and the patient’s individual risk factors, such as immunocompromise or recent exposures to healthcare environments [41]. The 2021 Surviving Sepsis Campaign guidelines on initial empiric antibiotic therapy selection suggest initiating broad-spectrum coverage within one hour of recognizing the signs and symptoms of sepsis, but then subsequently tailoring to patient-specific culture results and susceptibilities, as well as local antibiograms [42].

Empiric coverage for Gram-negative bacteria is often considered standard and uniform in the treatment of sepsis. These pathogens can release endotoxins derived from components of their cell wall, such as lipid A, which can be extremely immunogenic in eliciting massive inflammatory response in the form of cytokines (e.g., TNF-alpha) [43]. Additionally, pathogen-associated molecular patterns (PAMPs), which are breakdown products of microorganisms, have also been investigated both for their diagnostic and therapeutic utility, especially in the setting of culture-negative sepsis [44]. Commonly used antibiotics to treat Gram-negative sepsis include piperacillin-tazobactam, fluoroquinolones such as levofloxacin, cephalosporins such as ceftazidime and cefepime, as well as carbapenems such as meropenem [45]. Gram-negative coverage should include targeting *Pseudomonas aeruginosa*, a Gram-negative bacillus associated with high rates of antibiotic resistance and mortality [46]. Due to intrinsically high rates of antibiotic resistance in Gram-negative bacteria such as *Pseudomonas* and *Enterobacteriaceae*, the Infection Disease Society of America (IDSA) supports using double-agent combination coverage for Gram-negative organisms in those with septic shock [47] until patient-specific culture and susceptibility results return [48]. Additionally, for patients who are at high risk of methicillin-resistant *Staphylococcus aureus* (MRSA) infection either due to positive cultures in the past or recent exposure to the healthcare setting, empiric coverage for MRSA is recommended until definitive cultures result [49].

Once the organism and source of infection can be identified, source-control in the form of surgery and/or debridement or removal of lines/drains/indwelling catheters should be performed and all antimicrobials should be tailored and deescalated as soon as possible to avoid iatrogenic damage [42]. Among the various side effects of prolonged antibiotic use, one lesser-appreciated mechanism that may further complicate hemodynamic function in the setting of comorbid sepsis and CS is that carrier solutions for IV antibiotics can contain large amounts of sodium [50]. For example, with typical dosing, IV vancomycin can deliver an astounding 3540 mg of sodium over 24 h of therapy, while IV piperacillin-tazobactam will add another 2280 mg over the same duration [51]. The resulting volume expansion from this salt overload can incite pulmonary edema, cardiorenal syndrome, and other end-organ dysfunction, especially in the setting of existing cardiac dysfunction. Though appropriate antimicrobials are an essential therapy in sepsis, their ongoing judicious use is particularly important when managing comorbid CS.

### 5.2. Fluid Resuscitation

Optimal fluid resuscitation is an important component of sepsis care, as many outpatients present for medical care with hypovolemia due to low per oral intake and increased losses from fever-induced perspiration, which can be further complicated by massive inflammation-induced low vascular tone and leakage [52]. Until recently, most guidelines have recommended aggressive resuscitation with crystalloids in sepsis, with a 30 mL/kg of body weight initial bolus to be followed by maintenance fluids with the goal of achieving a consistent MAP > 65 mmHg [53]. However, contemporary meta-analyses of large RCTs have demonstrated that lower crystalloid resuscitation volumes did not lead to worse outcomes, raising doubt about the ideal fluid volume for sepsis management [54,55]. Thus, the current 2021 Surviving Sepsis guidelines cite insufficient evidence to make a recommendation about a liberal vs. restrictive fluid strategy in the first 24 h of resuscitation [49].

A longstanding debate exists about whether the use of colloids (e.g., albumin) for fluid resuscitation would improve outcomes compared to crystalloids. Only 25% of every 1 L of administered crystalloids, as opposed to nearly 50% of administered colloids, remains in the plasma after distribution to various intracellular and extracellular compartments. Additionally, the half-life of infused crystalloids vs. colloids in circulation is only around 20–40 min vs. 2–3 h, respectively [56,57]. Multiple RCTs (e.g., SAFE, CRISTAL, ALBIOS) compare the efficacy of crystalloids versus colloids for resuscitation in the ICU; however, none of them demonstrated a significant difference in mortality or renal outcomes [56,58,59]. In a similar vein, colloids are commonly used for post-cardiac surgery volume resuscitation, despite its higher costs and the lack of evidence for its superiority over crystalloids [60]. Notably, some of these studies’ conclusions are limited by their generalization of distinct types of colloid solutions as well as their use of low-concentration colloids. Recently, a meta-analysis of over 5000 patients with septic shock did show improved 90-day outcomes with the use of 20% albumin vs. crystalloid resuscitation, calling for further prospective studies [61,62].

Another consideration for fluid choice in resuscitation is choosing between different crystalloid solutions (Table 2). While normal saline, which contains 154 mEq of sodium and an equivalent amount of chloride, may be the most widely used option, recent clinical data support more “balanced” solutions, such as lactated ringers and Plasma-Lyte, in most clinical situations. As compared to normal saline, these fluids are comprised of electrolyte concentrations more physiologically similar to plasma [63]. This typically means lower levels of sodium and chloride, and the addition of 4–5 mEq/L of potassium, along with a buffer component (e.g., lactate) that is eventually metabolized to bicarbonate. Randomized controlled trials comparing the use of normal saline to “balanced” crystalloids have shown mixed results, with the SMART [64] and SALT-ED [65] trials showing a slight benefit for Ringer’s lactate, and BASICS [66] and PLUS [67] demonstrating no difference in mortality or renal outcomes. Proponents of the use of “balanced” crystalloids cite the high chloride load as a risk factor for hyperchloremic metabolic acidosis. Additionally, this chloride load delivered to the macula densa can be a major contributor to acute renal injury, as the tubulo-glomerular feedback causes afferent vessels to vasoconstrict [68]. Meanwhile, supporters of normal saline point to the added potassium of “balanced” crystalloids as potentially exacerbating hyperkalemia in at-risk patients, but this myth has been largely dispelled in follow-up studies [69].

For patients with comorbid sepsis and CS, frequently reassessing volume status to determine the need for fluid resuscitation or post-resuscitation fluid restriction is important. The CLOVERS study randomized patients with sepsis-related hypotension to either a restrictive or a liberal fluid management strategy and found no differences in 90-day mortality between the two cohorts [70]. Ultimately, the decisions surrounding fluid administration in comorbid sepsis and CS can be challenging, but starting with smaller fluid boluses and determining future needs with frequent reassessments based on exam and objective data is a prudent approach.

### 5.3. Vasopressors

Vasopressor use has been a cornerstone in the management of septic shock to address low vascular resistance, hypotension, and subsequent poor organ perfusion secondary to massive inflammation. In order to maintain the MAP target of ≥65 mmHg suggested by the 2021 Surviving Sepsis campaign [42], different vasoactive agents can be escalated or used in conjunction with one another. Norepinephrine is the most used agent and is considered first-line by experts and guideline consensus. Its activity on both alpha- and beta-adrenergic receptors contributes to not only increased vascular tone but also myocardial contractility. Vasopressin is a second-line agent often added when the MAP goal cannot be attained via norepinephrine administration alone, or to help reduce the catecholaminergic load that can potentially trigger problems such as cardiac dysrhythmias [70]. Vasopressin, or the antidiuretic hormone (ADH), acts on V1 and V2 receptors to increase vascular tone and fluid retention in the kidneys, respectively. For patients with CS requiring inotropic agents (e.g., milrinone, dobutamine) that have concomitant distributive shock secondary to sepsis, up-titrating vasopressor support is often necessary, and inotropic agent use should be optimized as well. Epinephrine is often used as a dual inotrope and vasopressor to help minimize norepinephrine requirements. Epinephrine’s strong beta-adrenergic agonism and alpha agonism make it useful for cardiac dysfunction, though when combined with norepinephrine, the risk for catecholamine overload increases. Specifically, the risk for life-threatening dysrhythmias and organ ischemia secondary to significant vascular tone increases with prolonged, combined use of such agents. Additionally, overuse of adrenergic agents may increase pulmonary vascular resistance, which can contribute to decompensation in patients with existing RV dysfunction. Because overuse of adrenergic agents can increase mortality in patients with CS [71], judicious use of the lowest dose and shortest duration possible is strongly recommended. Historically, dopamine has been widely used for shock due to its interesting dose-dependent dopaminergic and adrenergic receptor agonism; however, comparison studies against norepinephrine have shown more frequent adverse events such as dysrhythmias and increased mortality in patients with CS [72]. Newer inotropes such as levosimendan are also under active investigation for their efficacy and safety profiles compared to traditional agents [73], though no mortality benefit supports its use in lieu of standard therapies in septic shock [74]. The role of these newer agents will need to be studied further before recommendations can be reliably given.

Another particularly interesting agent worthy of discussion exerts its effects on the renin–angiotensin–aldosterone system (RAAS)—angiotensin II (AT-II). The RAAS has been a key pharmacological target in the treatment of hypertension, renal disease, and prevention of adverse cardiac remodeling, yet despite >30 years of clinical use, its role in the management of shock has been, until recently, overlooked [75]. A 2017 RCT demonstrated that AT-II effectively raised MAP by ≥10 mmHg from baseline or to ≥75 mmHg in patients with vasodilatory shock who required >0.2 μg/kg/min of norepinephrine infusion, compared to placebo [76]. AT-II improved the SOFA score at 48 h without increasing adverse events compared to placebo. A review of 24 studies involving the use of AT-II also demonstrated that it effectively increased MAP by an average of 23.4% in patients with circulatory shock [77]. Ultimately, pharmacological therapy targeting the RAAS pathway may be a powerful tool alongside adrenergic-modifying agents to treat shock.

### 5.4. Positive-Pressure Ventilation

Patients with sepsis often develop life-threatening respiratory compromise, either through direct infection of the pulmonary parenchyma (e.g., pneumonia) or through inducing acute respiratory distress syndrome (ARDS), where inflammation leads to massive pulmonary capillary leakage and alveolar collapse. These patients often need rapid up-titration of non-invasive or invasive positive-pressure ventilation (PPV) [78]. While PPV crucially supports alveolar expansion and oxygen exchange across the alveolar membrane, it also exerts varying hemodynamic effects on the left (LV) and right ventricle (RV). Generally, LV function benefits from PPV in the form of afterload reduction resulting from the baroreceptor reflex response to aortic compression [79]. However, PPV increases both intrathoracic pressure and pulmonary vascular resistance (PVR) [80]. These hemodynamic impacts become extremely important in the setting of RV dysfunction. The increase in intrathoracic pressure can decrease the amount of venous return, leading to lower RV preload. Concurrently, PPV can cause alveolar overdistension, which in turn compresses pulmonary vasculature, leading to increased PVR and thus increased RV afterload. Together, reduced RV preload and increased RV afterload can exacerbate underlying RV dysfunction, and significantly increase its work and oxygen demand [81]. Subsequently, severe RV dysfunction can lead to an insufficient supply of LV preload, inducing left-sided dysfunction and worsening cardiac output, eventually descending into a hemodynamic crisis. Thus, a delicate balance between ventilation, oxygenation, and perfusion must be carefully monitored and maintained [79].

### 5.5. Mechanical Circulatory Support

Temporary mechanical circulatory support (MCS) devices improve systemic perfusion, allow time for myocardial recovery from injury, and either serve as a bridge to recovery or as a bridge to a more definitive intervention (e.g., durable device, cardiac transplantation) [82]. The rapid evolution in MCS in recent years has provided newer options for CS patients who are refractory to fluid resuscitation and vasopressor therapy [83]. Right-sided univentricular support devices typically help circumvent the failing RV, thus improving LV preload and systemic perfusion. Examples of RV MCS devices include the Impella RP Flex and the Protek Duo [84]. Left-sided devices (e.g., intra-aortic balloon pumps (IABP), Impella, TandemHeart series) either directly contribute to cardiac output or increase coronary perfusion to the LV by improving diastolic coronary flow [82] Meanwhile, biventricular support can be achieved through venoarterial extracorporeal membrane oxygenation (VA-ECMO), biventricular assist devices (e.g., BiPellas, Impella/Protek, CentriMag surgical BiVADs), or the total artificial heart (TAH) [85]. Specifically, VA-ECMO is increasingly utilized as a bridge to definitive therapy in the form of either transplantation or long-term LVAD for patients with a cardiac index <2 L/min/m^2^ with lack of myocardial recovery, low MAP despite vasopressors, and other MCS modalities [86]. Despite high absolute mortality rates (i.e., 50–70%) for this patient cohort, VA-ECMO use in patients with MI-induced CS did not improve clinical outcomes [87,88]. Waiting to initiate VA-ECMO until patients with severe CS clinically deteriorate may yield similar outcomes to initiating it shortly after diagnosis. In fact, a recent large RCT showed that among patients with acute myocardial infarction complicated by CS, extracorporeal life support did not improve 30-day mortality compared to standard medical therapy [89,90]. Despite these findings, VA-ECMO remains an important tool in cardiogenic shock, and prudent clinical decision making is warranted to help identify patients who may benefit the most from VA-ECMO as a bridge to definitive therapy.

While MCS may not be indicated for primary septic shock, with careful consideration of risk and benefits, it can serve as a useful tool to manage refractory mixed vasodilatory and cardiogenic shock [91]. In patients developing sepsis or septic shock while already on MCS, a contingency plan should be made regarding if and when to escalate the level of mechanical support. For example, after a thorough risk–benefit analysis, a patient with only RV support can be considered for a temporary LV device if hemodynamics worsen with sepsis [92]. Similarly, patients on VA-ECMO should be considered for flow parameter up-titration if a mixed shock scenario develops [92]. Notably, in a recent retrospective analysis exploring the use of temporary MCS for sepsis-related cardiogenic shock, only the use of IABP and VADs was associated with a lower risk of in-hospital mortality [93]. Specifically, among patients with septic shock and acute MI, MCS failed to demonstrate a survival benefit. Though animal models are under investigation [94], further prospective, randomized control trials are needed to further elucidate outcomes of temporary MCS use in patients with comorbid septic and cardiogenic shock.

Another controversy surrounding the use of mechanical cardiac support in septic patients centers around the potential risk of introducing a new potential source or nidus of infection [91]. First, sepsis is not an absolute contraindication for the initiation of MCS [95]. As independent factors, septic shock and CS already each confer a high risk for mortality, and, when present together, they contribute to even more rapid decompensation [96]. Thus, the hemodynamic benefit that comes with the judicious use of mechanical support often outweighs the potential risk of a new device-related infection [97].

Finally, initiating different MCS, especially ECMO, can introduce both hemolysis and thrombotic risk, often secondary to the shearing stress exerted on red blood cells and platelets, thus warranting empiric anticoagulation [98,99]. Without large-scale, prospective investigation assessing how to optimally balance the bleeding and thrombotic risks of MCS use in patients with comorbid septic and cardiogenic shock, any risk–benefit discussion must include this. Whether the hemodynamic benefit provided by MCS outweighs the increased risk of major hemorrhage or venous thromboembolism should be an individualized decision for every patient.

## 6. Conclusions and Future Outlook

Sepsis management in the CICU is challenging, nuanced, and rapidly evolving due to the steady influx of new evidence on fluid resuscitation and vasopressor use, as well as the advancing MCS technologies. Artificial intelligence-guided screening and management tools for sepsis have also been undergoing active modeling and testing, though these initial applications should always be approached with equal parts optimism and caution.

## Figures and Tables

**Table 1:** Classic Hemodynamic Profiles of Discrete vs. Mixed Shock Etiologies.

Etiology of Shock	Cardiac Index (CI)	Systemic Vascular Resistance (SVR)	Central Venous O_2_ Saturation (ScVO_2_)	Central Venous Pressure (CVP)
Cardiogenic	Decreased	Increased	Decreased	Increased
Distributive	Increased or normal	Decreased	Increased	Decreased or normal
Hypovolemic	Decreased	Increased	Decreased	Decreased
Obstructive	Decreased	Increased or normal	Decreased or normal	Increased
Mixed Cardiogenic & Distributive	Decreased or variable	Decreased or variable	Variable	Variable

**Table 2:** Electrolyte and Osmolality Statistics of Common Resuscitation Fluids as Compared with Plasma.

Fluid Type	Sodium-Na (mEq/L)	Chloride-Cl (mEq/L)	Potassium-K (mEq/L)	Osmolality (mosm/L)
Plasma	140	103	4	290
Normal Saline (0.9%)	154	154	0	308
Lactated Ringers	130	109	4	273
Plasmalyte	140	98	5	294
Colloids (e.g., 25% albumin)	145	Dependent on diluent	Dependent on diluent	~300

## Data Availability

Not applicable.
